# Metacognitive therapy and work-focus for patients with depression, anxiety or comorbid depression and anxiety on sick leave: a single-centre, open-label randomised controlled trial

**DOI:** 10.1016/j.eclinm.2025.103613

**Published:** 2025-11-06

**Authors:** Ragne G.H. Gjengedal, Marit Hannisdal, Kåre Osnes, Silje E. Reme, Adrian Wells, Roland Blonk, Hilde D. Lending, Sverre U. Johnson, Suzanne E. Lagerveld, Frederick Anyan, Hans M. Nordahl, Romée B.T.W. Gerritsen, Marianne T. Bjørndal, Danielle Wright, Kenneth Sandin, Kjersti S. Bjøntegård, Jørund Schwach, Odin Hjemdal

**Affiliations:** a Division of Mental Health and Substance Abuse, Diakonhjemmet Hospital, Oslo, Norway; b Department of Psychology, University of Oslo, Oslo, Norway; c Division of Psychology and Mental Health, School of Psychological Sciences, Faculty of Biology, Medicine and Health, School of Health Sciences, University of Manchester, Manchester, United Kingdom; d Tilburg University, Tilburg, the Netherlands; e The Dutch Institute of Employee Benefit Schemes, Amsterdam, the Netherlands; f Department of Psychology, Norwegian University of Science and Technology, Trondheim, Norway; g Division of Psychiatry, Department of Mental Health, Norwegian University of Science and Technology, Trondheim, Norway; h Oslo University Hospital, Oslo, Norway; i The Norwegian Psychological Association, Oslo, Norway

**Keywords:** Depression, Anxiety, Sick leave, Work focus, Metacognitive therapy

## Abstract

**Background:**

Employment is a key determinant of health, but mental health treatments have limited success on return-to-work (RTW) in depression and anxiety. We investigated the effectiveness of metacognitive therapy combined with work-focused components (MCT + WF) to improve RTW and reduce anxiety and depression in patients on sick leave.

**Methods:**

This single-centre, open-label, randomised controlled trial was conducted at an outpatient clinic (Diakonhjemmet Hospital) in Norway. Eligible patients were adults on sick leave with depression and/or anxiety. Patients with severe mental disorder or substance abuse were excluded. Participants were randomly assigned using computer-generated block randomisation, stratified by gender and percentage of sick leave, to receive either immediate MCT + WF or delayed MCT + WF after 8–12 weeks on a waitlist. Outcome assessors were blinded. Primary outcomes were depression (BDI-II), anxiety (BAI), and RTW at 12 weeks. Sick leave data were obtained from a national registry and self-report; symptoms were self-reported. Analyses followed the intention-to-treat principle, including all randomised participants. This trial was registered with ClinicalTrials.gov, NCT03301922.

**Findings:**

Between Sept 11, 2017, and Nov 17, 2020, 236 patients were enrolled and randomly assigned to immediate MCT + WF (*n* = 121) or waitlist (*n* = 115). At 12 weeks, logistic regression of registry data showed significantly higher RTW in the immediate MCT + WF group (39%; 47/121) than in the waitlist group (20%; 23/115; OR = 2.39, 95% CI 1.32–4.32; p = 0.0040), consistent with self-reported RTW (42% (51/121) versus 18% (20/114); OR = 3.44, 95% CI: [1.87, 6.35], p < 0.0001). Multilevel models revealed greater reductions in anxiety (time × group interaction coefficient = −8.35, 95% CI –10.61 to −6.09; p < 0.0001) and depression (−10.84, 95% CI –13.25 to −8.44; p < 0.0001) for immediate MCT + WF versus waiting. No serious adverse events were reported during the study.

**Interpretation:**

Immediate MCT + WF significantly improved RTW and reduced symptoms of depression and anxiety compared with waiting. Generalisability may be constrained by Norway's welfare system; strengths include registry data and naturalistic outpatient setting. The favourable outcomes suggest that MCT + WF could be integrated into mental health care.

**Funding:**

The study was funded by the South-Eastern Norway Regional Health Authority and DAM foundation-Mental Health, Diakonhjemmet Hospital.

**Research in context**

**Evidence before this study**

We searched PsycINFO, MEDLINE and Embase using the main search terms “randomised controlled trials”, “common mental disorder”, “depression”, “anxiety”, “therapy” “work focus”, “work interventions” “return-to-work”, “sick leave”, and “absence” and identified 5 systematic reviews and meta-analyses published before September 16th, 2025. They investigated psychological clinical interventions on sick leave and psychiatric symptoms in common mental disorders (CMD), including stress-related disorders. Most clinical interventions had small or non-significant effects on sick leave and symptoms. A Cochrane review that included a longitudinal analysis found interventions for depression did not significantly increase return-to-work rates at one-year. Additionally, work interventions alone may increase the rates of sick leave among people already on sick leave. Although these reviews indicate it is essential to incorporate work interventions into therapy to increase work participation for individuals with CMD, the characteristics or components of effective work interventions are poorly defined. In addition, a recent WHO guideline recommends that return-to-work programs should include both work-directed and mental healthcare interventions, although there is uncertainty in the evidence base supporting this recommendation. Integrated vocational rehabilitation with combinations of two or more practitioners or complex RTW programs were excluded. We did not identify any other studies of individual therapies with work interventions for CMD with return-to-work as an outcome that were not already included in previous reviews.

**Added value of this study**

To our knowledge, this is the first randomised clinical trial to investigate the effect of metacognitive therapy combined with integrated work focus (MCT + WF) for patients on sick leave with depression and anxiety in a pragmatic, real-world study at an outpatient clinic. We compared a group that received MCT + WF immediately after referral and a waiting list group that represents usual practice in mental healthcare. Immediate MCT + WF led to significantly improved outcomes in terms of both return-to-work rates and symptom recovery at post-treatment compared to the waiting list group, and these effects were sustained at 12-months follow-up.

**Implications of all the available evidence**

Evidence from previous systematic reviews and meta-analyses suggests that some psychological treatments for CMD that include work interventions may have an impact on sick leave, albeit with low effect sizes. However, not all studies reported effects on the return-to-work rates and some work interventions had a negative impact, suggesting that the optimal combinations of therapy and work interventions remain unclear. The current study provides evidence for the effectiveness of MCT with embedded work focus modules compared to a regular waiting list in terms of improved return-to-work rates and symptom recovery among patients with depression or anxiety on sick leave. Limitations such as the generous Norwegian welfare context and a waitlist control may influence generalisability. To expand generalisability replications across different healthcare and welfare systems and comparison with active conditions are required.

## Introduction

Depression and anxiety, common mental disorders (CMD) that severely impact quality of life and the global burden of disease,^1^ frequently co-occur, have high relapse rates, and can lead to persistent and severe impairment throughout life.^2^ CMD also contribute substantially to sick leave and work disability and impose widespread economic implications and public health concerns.^3^ Global productivity losses alone due to CMD have been estimated at US$ 1 trillion per year and are forecast to reach US$ 16 trillion by 2030.^4^

Sick leave rates in Norway are among the highest in the OECD.^5^ Despite a generous national sick leave scheme intended to promote an inclusive work environment, long-term sick leave rates are particularly high for individuals with CMD. Absence from work due to CMD potentially deprives individuals of activities and social support that contribute to well-being and perpetuates a cycle of poorer mental health, stigma, and social inequality.^6^ New approaches to improve return-to-work (RTW) rates are urgently needed, as a substantial body of evidence confirms suitable employment can benefit mental health and overall well-being in individuals with depression or anxiety.^7^

Research on psychotherapy for CMD has reported small to nonsignificant effects on sustainable RTW.^8^^,^^9^ Thus, there is a pressing demand to develop effective treatments to help patients with CMD RTW. Such treatments need to be evaluated in realistic clinical settings and address both symptomatic and functional recovery to ensure high ecological validity and generalisability.^6^^,^^10^ Current challenges in regular mental healthcare include prolonged waiting lists and limited access to treatment; these issues are particularly critical, as delayed or no treatment may increase the risk of mental health deterioration and long-term workforce exclusion.^6^

In addition, previous studies suggested psychotherapy has limited effectiveness for CMD, with only approximately half of patients achieving symptomatic recovery.^10^ However, recent evidence suggests Metacognitive Therapy (MCT) may lead to improved outcomes and reduced relapse rates.^11^, ^12^, ^13^ MCT, a transdiagnostic treatment that aims to handle multi-morbidities in a single treatment package, focuses on goal-directed processing and biases in the regulation of thinking and coping that maintain emotional disorders and interfere with personal goal attainment. Thus, MCT could represent a useful approach to improve mental health and RTW.^13^

Both mental health and workplace factors influence the duration and nature of sick leave for patients with CMD. WHO guidelines on work-directed care state treatment for patients on sick leave should be effective for their mental disorder(s) and also support a return to a meaningful work.^6^ However, previous randomised controlled trials (RCT) investigating work-directed treatment achieved inconsistent and low effect sizes for symptoms and limited to no effect on RTW rates.^8^^,^^9^^,^^14^^,^^15^ Moreover, the work-directed interventions in these studies were heterogeneous, not clearly defined, and delivered by a mixture of therapists and labour experts.^14^

Nevertheless, a recent quasi-controlled study from the Netherlands reported that integration of cognitive therapy (CBT) and work focus, primarily for adjustment disorder, had positive effects on both symptoms and sick leave.^16^ The work focus included assessing each patient's work situation, examining factors that may facilitate or hinder RTW, and planning a gradual return.^16^ Moreover, the CBT and work focus were delivered by the same psychotherapist, which increased the relevance of the treatment for both symptoms and RTW.

The poor outcomes associated with previous RCTs for CMD could be due to the utilisation of therapies with modest effects and poor conceptual integration between the work interventions and the underlying treatment model. MCT focuses on self-regulation and decision-making strategies and is guided by a specific model of when and how these processes are diminished, thus can be applied to both coping with emotions and the challenge of returning to work. In addition, the stronger theoretical and conceptual consistency between the therapeutic mechanisms and the work-focused content might increase the effectiveness of treatment in relation to both RTW and symptom recovery.

Given the need to evaluate and implement effective therapy for CMD and improve RTW rates, we conducted a pragmatic RCT to test the effects of MCT and work focus (MCT + WF) for patients with CMD on sick leave. Work focus was conceptually integrated with the underlying MCT treatment model.^17^

As delayed treatment is common practice in standard mental health outpatient clinics, we compared the impact of immediate MCT + WF to a waiting list group. We hypothesised that participants receiving immediate MCT + WF would exhibit significantly greater improvement in the four co-primary outcomes: anxiety, depression, and RTW rates assessed through a national sick leave registry and self-reports, compared to the patients in the waiting list group. We also exploratively assessed whether immediate MCT + WF compared to the waitlist led to greater improvement in several secondary outcomes, specifically metacognitions, health-related quality of life, RTW self-efficacy, resilience, and subjective health complaints. Finally, longitudinal analyses were conducted during one-year follow-up after treatment for both groups to explore the changes in RTW rates, depression, anxiety, metacognitions, health-related quality of life, RTW self-efficacy, resilience and subjective health complaints. Additionally, we estimated productivity costs and quality-adjusted life years.

## Methods

### Study design and participants

The study was conducted in a routine psychiatric outpatient clinic at Diakonhjemmet Hospital in Oslo, Norway that is a part of the national healthcare service. Patients are referred by general practitioners (GP). In Norway, sick leave is certified by GPs. The Norwegian Labor and Welfare Administration (NAV) covers 100% of the worker's salary for up to 52 weeks of sick leave; the first 16 days of sick leave are paid for by the employer. Waiting times after referral are common for patients with CMD in this healthcare system.

In their assessment session, patients were invited to participate in the study and were provided with a written informed consent form. The recruitment was consecutive for all patients referred to the clinic. The patients were informed about the research project by the assessor, a clinical psychologist. Patients could withdraw at any time without giving a reason or facing consequences for their continued treatment. The study was preregistered at clinicaltrials.gov (NCT03301922) and approved by the Norwegian Regional Committee for Medical and Health Research Ethics (2016/1869 REK) and the hospital's data protection officer. The trial protocol was previously published.^18^ Proper reporting of the study results was ensured by adhering to the Consolidated Standards of Reporting Trials (CONSORT) reporting guidelines for randomised trials.

Inclusion criteria were a diagnosis of major depressive disorder, anxiety disorder, or comorbid depression and anxiety, as defined by the MINI International Neuropsychiatric Interview, 6.0.0 (MINI) and a clinical assessment based on the 10th edition of the International Statistical Classification of Diseases and Related Health Problems (ICD-10); age 18‒67-years-old; on partial (20–90%) or full sick leave; and literacy in Norwegian. Exclusion criteria were current psychotic disorders, including bipolar disorder; current substance abuse; acute suicidality; severe depression states requiring inpatient treatment; and cluster A/B personality disorders. Patients with these disorders get specialised treatment in other units. Assessments were undertaken in accordance with the national guidelines and clinical practice for specialised mental healthcare services in Norway.^19^

### Randomisation and masking

After their initial assessment session, eligible patients were randomly assigned to the treatment group, which received immediate MCT + WF (*n* = 121), or the regular waiting list group (*n* = 115), who only received follow-up from their GP during the waiting list period if they had medical needs. At the end of waiting (either 8 or 12 weeks), all patients in the waiting list group also received MCT + WF. The Norwegian National Priority Guidelines state patients with moderate depression are entitled to receive specialised mental healthcare within 56 days of referral (8 weeks) and patients with functionally disabling anxiety are guaranteed care within 84 days (12 weeks). Thus, stratified randomisation dependent on diagnosis was conducted to adhere to the priority guidelines: patients with moderate depression were randomised to immediate MCT + WF or an 8-week waiting list; patients with primary anxiety disorders were randomised to immediate MCT + WF or 8- or 12-week waiting lists.

A web-based program (WebCRF) provided by the Unit for Applied Clinical Research at the University of Science and Technology (NTNU) was used for random allocation. Randomisation was block-stratified based on gender and percentage of sick leave and conducted by independent administrative staff not involved in clinical care.

### Procedures

Participants were recruited between Sept 11, 2017 and Nov 17, 2020. The first study-related treatment session was delivered on Sept 26, 2017. The study continued during COVID-19 pandemic-imposed restrictions, although at a slower inclusion pace. Nine MCT-registered therapists trained at the Metacognitive Institute and specially trained to perform work focus delivered the therapy. The same therapist delivered MCT and WF for each patient; planned treatment was 12 sessions of combined MCT + WF, once per week. Therapists received regular group supervision from MCT and work focus specialists. Patients were offered up to five booster sessions after treatment to consolidate knowledge and strategies acquired during MCT + WF.

At pre- and post-treatment, the MINI 6.0.0 was used to screen for psychiatric diagnoses via structured interviews by an independent clinician not involved in delivering treatment and blinded to trial allocation.

The therapists’ self-delivered treatment adherence checklist scales for the MCT and WF interventions are described in Appendix (pp 3–8).

#### Metacognitive therapy (MCT)

MCT was delivered in accordance with a treatment manual.^13^ For further details see Appendix (p 2). The MCT case-formulation was selected according to the patient's primary disorder. MCT targets cognitive attentional syndrome (CAS), a persistent style of unhelpful thinking and coping characterised by worry, rumination, and threat-monitoring central to depression and anxiety disorders. MCT aims to modify metacognitions, i.e., beliefs about thoughts and mental regulation strategies that drive CAS, which is postulated to maintain CMD. For further details, see Wells (2009).^13^

Patients with CMD on sick leave often worry and ruminate about work-related issues, such as performance, interactions with supervisors, and colleagues’ perceptions and responses. During MCT, maladaptive coping strategies such as avoidance, reassurance-seeking, procrastination, and excessive threat monitoring of performance in relation to work or work-related topics were addressed. For example, therapists worked with clients to understand and replace unhelpful self-regulatory strategies with alternative strategies that increase flexibility and choice in terms of thinking and behaviour when managing emotions and preparing for RTW. Thus, work issues can be introduced and discussed at different stages of therapy in a manner that remains consistent with the therapeutic mechanisms of MCT and embeds work-focused content within the model.

#### Work-focused components

The work-focus (WF) aligned with the MCT treatment model (Appendix pp 3–7) and included: (1) an initial assessment of the work situation, including job type, relationships with colleagues, and interplay between work and mental health, considering the potential benefits and drawbacks of sick leave; (2) development of a RTW plan to address facilitating factors and potential barriers, including the possible need to change employment; (3) cooperating with the responsible certifying GP; and (4) development of a communication plan to guide the individual's process of disclosure at the workplace. WF components were flexibly tailored to each patient's needs. No modifications were made to the disorder-specific MCT protocol.

### Outcomes

The four co-primary outcomes were changes in symptoms of depression, changes in the symptoms of anxiety, the RTW rates at 12 weeks after assessment in both groups based on sick leave data obtained from the NAV data registry, and self-reported sick leave post-treatment in the immediate MCT + WF group (i.e., 12th treatment session, or last session if therapy was shorter) versus post-waiting in the waiting list group. This time frame adheres to national priority guidelines, which states that all patients with anxiety and mild depression should receive treatment in specialised mental healthcare within 12 weeks. Depression and anxiety symptoms were evaluated using the 21-item self-reported Beck Depression Inventory II (BDI-II)^20^ and Beck Anxiety Inventory (BAI).^21^ The RTW rates based on NAV registry data indicate whether participants were on sick leave or not, while the self-reported RTW rates reflect whether participants were engaged in full-time work versus less than full-time.

Secondary outcomes were measured at pre- and post-treatment for the MCT + WF group and pre- and post-waiting for the waiting list group using the self-reported RTW Self-Efficacy questionnaire (RTW-SE),^22^ Resilience Scale for Adults (RSA),^23^ Subjective Health Complaints questionnaire (SHC),^24^ Health-Related Quality of Life (EQ-5D-5L),^25^ Metacognitions Questionnaire 30 (MCQ-30),^13^ The Standardised Assessment of Personality Abbreviated Scale – Self report (SAPAS-SR),^26^ and Alcohol Use Disorder Identification Test (AUDIT).^27^ Patients with primary alcohol use disorders or personality disorders were not treated at this outpatient clinic. Therefore, to reduce patient burden, SAPAS-SR and AUDIT were collected only at pre- and post-treatment.

As exploratory analyses, we assessed the longitudinal changes in RTW rates measured as the percentage of sick leave in the registry data 12 months after assessment. Furthermore, we explored changes in depression and anxiety symptoms, and the secondary outcomes listed above between post-treatment in both groups, and at 6 and 12 months follow-up after treatment. Quality-adjusted life years was assessed using the EQ-5D-5L (Appendix pp10).

Demographic information and workplace bullying were assessed at initial assessment using the self-reported Negative Acts Questionnaire.^28^ In this study, we did not collect race or ethnicity data, as race is not routinely used as a classification in Norwegian health research, and there is no national registry collecting such information. We acknowledge that cultural or migration background could have served as a proxy measure. Instead, we collected detailed data on education level, which we believe captures important structural determinants of health.

### Choice of primary outcomes

Depression symptoms, anxiety symptoms, and RTW rates based on the registry data and self-reported data were assessed as co-primary outcomes. Chevance^29^ emphasises the importance of evaluating both symptoms and functional outcomes in clinical trials assessing treatments for CMD, and this focus was also highlighted by the user representatives’ group for this RCT. Therefore, the aim of this study was to evaluate both RTW outcomes and the changes in depression and/or anxiety symptoms, since the treatment needs to show effects for both symptoms and RTW to help patients effectively. We assessed RTW using two data sources: the national sick leave registry, as an objective measure with no missing data, and self-reported RTW data, which is commonly used in international studies in countries with no national registries. The BDI-II and BAI were used to assess depression and anxiety symptoms due to their reliability and validity in research and clinical settings.

### Statistical analysis

The power calculation for full RTW without sick leave was based on the minimum expected effect of the MCT + WF, as reported in a previous naturalistic follow-up study conducted at the same clinic.^17^ In that study, we observed a self-reported full RTW response rate of 0.41 in the intervention group compared to 0.26 in the wait-list control group, with a pooled standard deviation of 0.4. Therefore, 120 patients were required in each treatment group to have an 80% chance of detecting a significant difference in a dichotomous outcome variable (the self-reported and registry RTW data) at a one-sided significance level of 0.05.^18^ For the anxiety and depressive symptoms, 120 participants in each group would give a >99% chance of detecting an eight-point difference in the BDI-II and BAI scores between the immediate MCT + WF group and waiting list group using a pooled standard deviation of 6.95.

All analyses adhered to intention-to-treat (ITT) principles. SPSS 29.0 was used for descriptive and regression analyses. Chi-squared tests and independent t-tests were used to compare demographic and clinical characteristics. A multiple logistic regression model was used to compare RTW rates based on the national sick leave registry and self-reported sick leave between groups 12 weeks after assessment for national sick leave registry and for self-reported sick leave. All other analyses were performed in STATA IC version 17.0. Differences in symptoms between groups were assessed using planned contrast within multilevel modelling, which allows for estimation of changes in repeated measures over time despite missing data. For all the outcomes with missing data, Little's tests showed non-significant results, which indicate that the data were missing completely at random (p = 0.995).^30^ Therefore, missing data were handled using the full information maximum likelihood estimation. All models included time and group as fixed effects. For the primary outcomes, random intercept models were used. In the random coefficients model for secondary outcomes, we investigated the extent to which, after having received treatment, the gains in both groups were sustained or improved at 6 and 12 months. We computed a time by group effect to represent the difference in change over time between groups. The linear combination of coefficients function was used to create model-predicted means and all planned contrasts. Model-predicted means and SE values are presented for continuous variables at all timepoints from pre-treatment to follow-up.

Recovery rates were estimated using the Reliable Change Index (RCI) according to Jacobson and Truax (1991).^31^ The clinical cut-offs are based on norms from Seggar et al. (2002)^32^ and Gillis et al. (1995),^33^ which were 14 and 15 for the BDI-II and BAI, respectively. Change was classified using validated cut-off scores as ‘deteriorated’ if scores increased by ≥ 9 points; ‘unchanged’ if changed by < 9 points, ‘improved’ if decreased by ≥ 9 points but remained above the clinical cut-off, and ‘recovered’ if decreased by ≥ 9 points and scored above clinical cut-off before treatment and below after treatment.

We calculated within-group and controlled between-group effect sizes using Cohen's *d*. The productivity costs were estimated by multiplying the days of sick leave in each group by the average national wage, adjusting for differences in income between men and women in Norway (Appendix p 9).

### Role of funding sources

The funders of the study had no role in study design, data collection, data analysis, data interpretation, or writing of papers. The corresponding author had full access to the data and final responsibility for submitting the results for publication.

## Results

**Fig. 1:**
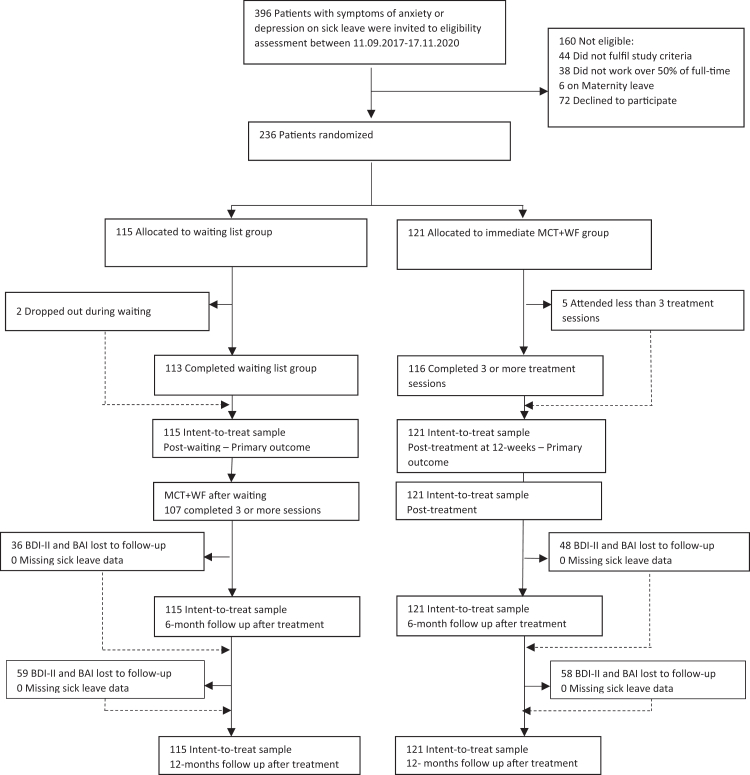
Consort flow diagram for the total ITT sample. MCT + WF = Metacognitive therapy + work focus.

Recruitment took place between Sept 11, 2017 and Nov 17, 2020. Participant flow and attrition rates are outlined in Fig. 1. Of the 396 patients assessed, 236 were eligible and consented to participate in the ITT sample: 115 (49%) were randomly assigned to the waiting list group and 121 (51%) to the immediate MCT + WF group. After the waiting list period, two (2%) patients did not attend treatment. In the immediate MCT + WF group, five (4%) participants attended fewer than three treatment sessions (Fig. 1).

Table 1 presents the patients’ baseline characteristics. Mean age was 38.5 (SD 9.7) years and there were more female participants (75%; 176/236). Consistent with successful randomisation, no significant differences in baseline characteristics or demographic features were observed between groups (Table 1). The most prevalent single MINI diagnosis pre-treatment was major depressive disorder, followed by generalised anxiety disorder (Table 2).

**Table 1:** Baseline characteristics and demographics for the total ITT sample.

	Waiting list group (*n* = 115)	MCT + WF group (*n* = 121)	p-value
**Gender**			
Male	27 (23%)	33 (27%)	p = 0.55
Female	88 (77%)	88 (73%)	
**Age, years**	39.7 (9.3)	37.4 (9.9)	p = 0.083^a^
**Education level**			
Primary school	0	5 (4%)	p = 0.097
Secondary school	17 (15%)	22 (18%)	
University college 1–4 years	50 (43%)	54 (45%)	
University >4 years	48 (42%)	40 (33%)	
**MINI Diagnosis**			
GAD	52 (45%)	59 (49%)	p = 0.60
Social phobia	9 (8%)	15 (12%)	p = 0.29
Panic disorder	24 (21%)	32 (26%)	p = 0.36
Major depressive disorder	92 (80%)	92 (76%)	p = 0.53
Comorbid depression and anxiety	49 (43%)	53 (44%)	p = 0.90
**Sick leave**			
Full sick leave	63 (55%)	59 (49%)	p = 0.37
Partial sick leave	52 (45%)	62 (51%)	
**Reported workplace bullying**	25 (22%)	20 (17%)	p = 0.33
**Relationship status**			
Married/cohabiting	69 (60%)	83 (69%)	p = 0.18
Living alone	46 (40%)	38 (31%)	
**Use of antidepressants in the year before participation**	13 (11%)	23 (19%)	p = 0.11

Data are mean (SD) and *n* (%). To explore group differences, we used Fisher's exact test for categorical data, and independent sample t-tests.

a For continuous data.

**Table 2:** Number of diagnoses based on the MINI International Neuropsychiatric Interview at pre-treatment and the end of treatment for the total ITT sample (N = 236).

MINI diagnosis	Pre-treatment	End of treatment		
	Diagnosis	Diagnosis	No diagnosis	Missing
Generalised anxiety disorder	111	3 (3%)	80 (72%)	28 (25%)
Social phobia	24	3 (13%)	16 (67%)	5 (21%)
Panic disorder	56	1 (2%)	33 (59%)	22 (39%)
Major depressive disorder	184	14 (8%)	112 (61%)	58 (31%)
Comorbid depression and anxiety	102	1 (1%)	71 (70%)	30 (29%)

Data are total number of diagnoses, *n* (%). No diagnosis for comorbid depression and anxiety means neither depression nor anxiety at post-treatment. Missing was attributed due to data collection being partly completed during the COVID-19 affecting the routines, and administrative errors at the clinic, or patient dropout.

No serious adverse events were reported during the study. The median number of sessions for the total ITT study sample (N = 236) was 10 (IQR: 7–13). In the total ITT sample, 6% (13/236) of participants dropped out of therapy, which was defined as less than three treatment sessions—which we considered minimal treatment. A total of 41 patients received a mean of 3.7 booster sessions in the year following treatment. Therapists used adherence checklists to self-report the content and interventions they used in each therapy session (Appendix pp 3–8).

For the self-reported symptom questionnaire, the response rates for the total sample were 97% (228/236) at end of treatment and 64% (152/236) and 50% (119/236) at 6 and 12 months, respectively (Fig. 1). There were no missing data for sick leave.

### Primary outcomes

#### RTW rates based on the national registry and self-reported data at 12 weeks after assessment

**Fig. 2:**
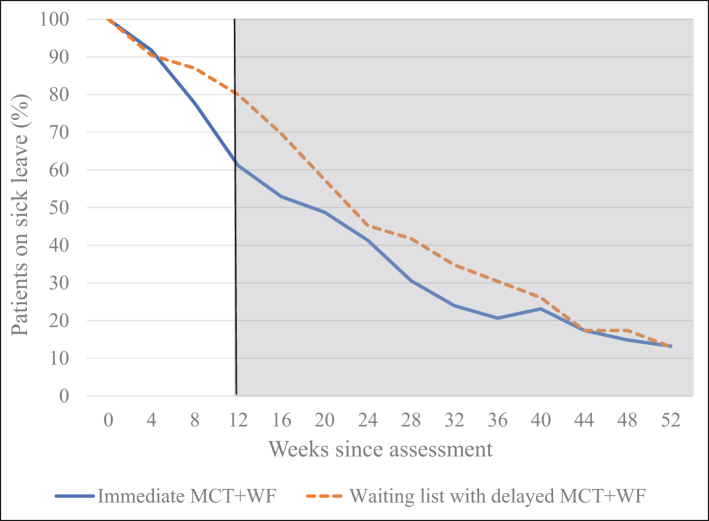
Percentage of patients on sick leave in the immediate MCT + WF group (*n* = 121) and waiting list group (*n* = 115) that received delayed MCT + WF between the assessment session and 52 weeks after assessment. The *x*-axis represents the 52 weeks after assessment. The *y*-axis shows the percentage of patients on sick leave based on national registry data for sick leave for the immediate metacognitive therapy + work focus (MCT + WF) group (solid blue line) and waiting list group that received delayed MCT + WF treatment (orange dotted line). The vertical black line illustrates the time point of analysis for the primary outcome (sick leave rates) for both groups at 12 weeks after initial assessment (regression analysis in Table 3). The grey area indicates the time interval when all patients had started to receive or had received MCT + WF treatment. Median number of sessions was 10 (IQR, 7–13) in both groups (*n* = 236).

To assess the effects of the MCT + WF treatment, we first compared the rates of full RTW (i.e., patients not on sick leave) using the national registry data at 12 weeks after assessment. A multiple logistic regression model adjusted for age, gender, and educational level showed significantly more patients in the immediate MCT + WF group (39%; 47/121) than the waiting list group (20%; 23/115) had returned fully to work at 12 weeks after assessment; this difference was significant (OR = 2.39, 95% CI [1.32, 4.32], p = 0.0040; Table 3 and Fig. 2).

**Table 3:** Multiple logistic regression analyses of full return-to-work with registry data at 12 weeks after assessment as a dependent variable between the immediate MCT + WF group (*n* = 121) and the waiting list group (*n* = 115), controlled for age, gender, and level of education.

	OR/Exp(B)	p-value	95% Cl
Age	0.99	0.42	[0.96–1.02]
Gender	1.35	0.36	[0.71–2.60]
Education level	0.65	0.18	[0.35–1.21]
Group	2.39	0.0040	[1.32–4.32]

Gender: 0 = female. Education was dichotomised as high (college/university) or low (primary or secondary school). Female, low education and waiting list group are reference categories.

We also assessed RTW using self-reported data. A multiple regression model of the self-reported RTW rates adjusted for age, gender and education level showed that significantly more patients in the immediate MCT + WF had returned fully to work (OR = 3.44, 95% CI [1.87, 6.35], p < 0.0001) post-treatment (i.e., 12th treatment session, or the last session if therapy was shorter; 42%; 51/121): compared to post-waiting in the waiting list group (18%; 20/114). Thus, the MCT-WF treatment increased RTW rates within a short period of time.

#### Anxiety and depression at the 12th treatment session versus end of waiting

Next, we analysed the changes in depression and anxiety symptoms between assessment and post-treatment (the 12th session or, if shorter, last session) for the immediate MCT-WF group (median sessions = 10, IQR 7–12) and after waiting for the waiting list group, when they had waited an average of 9.14 weeks (SD = 2.15, IQR 7.86–11.29). The planned contrast in the multilevel model analysis revealed that both BDI-II depression (time × group interaction coefficient = −10.84; 95% CI [−13.25, −8.44]) and BAI anxiety (time × group interaction coefficient = −8.35; 95% CI [−10.61, −6.09]) scores decreased significantly more in the immediate MCT + WF group than in the waiting list group (Table 4), indicating immediate MCT + WF treatment led to significantly greater reductions in anxiety and depression symptoms than waiting. The within-subject effect sizes (Cohen's *d*) for the immediate MCT + WF group were 1.54 for depression (BDI-II) and 1.11 for anxiety (BAI) compared to 0.36 and 0.28, respectively, for the waiting list group. The controlled effect size for the between-group differences from pre-treatment to end of treatment was 1.13 for depression and 0.77 for anxiety.

**Table 4:** Multilevel modelling with planned contrast of primary outcomes at the end of treatment/after treatment in the immediate MCT + WF group (*n* = 121) and post-waiting in the waiting list group (*n* = 115).

Primary outcome	*n*	Model-predicted means				
		Assessment (SE)	Post waiting/12 session (SE)	Time x group effect coefficient (p-value)	Time x group effect 95% CI	*d*
BDI-II						
Waiting list	115	28.27 (0.82)	24.93 (0.91)	−10.84 (<0.0001)	[−13.25, −8.44]	0.36
MCT + WF (12th session)	121	28.88 (0.79)	14.69 (0.89)			1.54
Group difference	236	0.61 (1.14)	−10.23^a^ (1.27)			
BAI						
Waiting list	115	20.32 (0.91)	17.52 (0.95)	−8.35 (<0.0001)	[−10.61, −6.09]	0.28
MCT + WF (12th session)	121	20.59 (0.89)	9.45 (0.92)			1.11
Group difference	236	0.27 (1.27)	−8.07^a^ (1.32)			

Primary outcomes were measured at the 12th session (or the last treatment session, if treatment was shorter) in the immediate MCT + WF group (median number of sessions = 10, IQR 7–12) and after a mean waiting period of 9.14 weeks in the waiting list group.

Reductions in the BDI-II and BAI scores indicate improvement.

a p < 0.05. BDI-II = Beck Depression Inventory II. BAI = Beck Anxiety Inventory.

At the end of treatment (median sessions = 10, IQR 7–13), 76% (90/119) of the immediate MCT + WF group had either recovered or exhibited improvements in the severity of their depression symptoms compared to 17% (19/109) of the waiting list group at the end of waiting. Moreover, 78% (71/91) of the immediate MCT + WF group had either recovered or exhibited improvements in the severity of their anxiety symptoms at the end of treatment compared to 28% (24/87) of the waiting list group at the end of waiting. In the immediate MCT + WF group, 2% (2/119) and 3% (3/91) of patients reported deterioration of depression or anxiety symptoms post-treatment compared to 3% (3/109) and 6% (5/87) of the waiting list group at the end of waiting (Table 5).

**Table 5:** Recovery rates for depression and anxiety measured with the BDI-II and BAI after treatment in the immediate MCT + WF group and post-waiting in the waiting list group, and for the total ITT sample (N = 236) at the end of MCT + WF treatment.

	*n*	Deterioration	No change	Improved	Recovered
**BDI-II**					
MCT + WF post-treatment	119	2 (2%)	27 (23%)	20 (17%)	70 (59%)
Waiting list post-wait period	109	3 (3%)	87 (80%)	15 (14%)	4 (4%)
Total sample post-treatment	228	2 (1%)	56 (25%)	37 (16%)	133 (58%)
**BAI**					
MCT + WF post-treatment	91	3 (3%)	17 (19%)	8 (9%)	63 (69%)
Waiting list post-wait period	87	5 (6%)	58 (67%)	10 (11%)	14 (16%)
Total sample post-treatment	178	3 (2%)	37 (21%)	15 (8%)	123 (69%)

Only participants who reported symptoms on or above the cut-offs for clinically significant levels of symptoms at pre-treatment were included in these analyses; therefore, the sample sizes for the recovery analysis are not the same as the group sizes. Patients were included in the analyses for both depression and anxiety symptoms if their assessment score met the respective cutoff score of 14 and 15 for the BDI-II and BAI, respectively.

BDI-II = Beck Depression Inventory II. BAI = Beck Anxiety Inventory.

### Secondary outcomes

First, we compared the secondary outcomes after the immediate MCT + WF group had completed the last treatment session (median sessions = 10, IQR 7–13) and after waiting/before the first treatment session for the waiting list group, after waiting an average of 9.14 weeks (SD = 2.15). Multilevel model analyses revealed significant improvements in RTW self-efficacy (RTW-SE) and the scores for resilience (RSA), subjective health complaints (SHC), quality of life (EQ-5D-5L), and metacognitions (MCQ-30) in the immediate MCT + WF group at the end of treatment compared to after waiting in the waiting list group. There were no significant between groups differences in change from pre-to post-treatment on SAPAS and AUDIT (Supplementary S1).

We also assessed the secondary outcomes 6 and 12 months after the end of treatment. Both groups demonstrated sustained improvements in the depression (BDI-II) and anxiety (BAI) symptom scores, as well as the RTW-SE, RSA, SHC, EQ-5D-5L, and MCQ-30 scores between end of treatment and 6 and 12 months (Supplementary S2). There were no significant differences in the majority of secondary outcomes after treatment between groups, except that the immediate MCT + WF group exhibited greater improvements in depressive symptoms and subjective health complaints at 12 months.

### Productivity cost analysis

Finally, we compared the costs up to the 12-month follow-up after initial assessment. The MCT + WF group had an average of 72 sick leave days, corresponding to a productivity cost of 22,718 euros (SD 18.20) per patient. The waiting list group had an average of 94 sick leave days and cost of 29,659 euros (SD 20.53), reflecting a mean difference of 22 sick leave days per patient between groups (t = 2.63, p = 0.0092). Consequently, the MCT + WF group experienced a significant benefit in terms of productivity costs (reflected in reduced sick leave), amounting to 6941 euros per patient over the 12-month period, which indicates that immediate MCT + WF was associated with large savings compared to delayed treatment. See Appendix (pp 9–10) for further treatment costs, sick leave calculations, and assessment of quality-adjusted life years.

## Discussion

Treatments that significantly improve mental health and increase RTW rates remain elusive. We conducted a pragmatic RCT to assess the impact of metacognitive therapy combined with work focus (MCT + WF) on symptom recovery and RTW rates among patients experiencing depression, anxiety or comorbid depression and anxiety on sick leave. The results indicated high rates of reliable improvements in anxiety and depression symptoms and recovery among the group that received immediate MCT + WF compared to the low rates observed during waiting. At the primary endpoint based on national sick leave registry data at 12 weeks after inclusion, 39% of patients in the immediate MCT + WF group had returned to work, compared to only 20% after waiting in the waiting list group. Consistent with these findings, self-reported sick leave showed that by the end of treatment in the immediate MCT + WF, 42% had returned fully to work, compared with 18% after waiting in the waiting list group.

In addition, comparison of the end of treatment and end of waiting scores favoured immediate MCT + WF over waiting across all secondary outcomes. However, after the waiting period, the waiting list group received MCT + WF and achieved equivalent effects during treatment as the immediate MCT + WF group. Moreover, in both groups, further reductions in sick leave rates and sustained symptom reduction were observed during the year after treatment.

Immediate MCT + WF also demonstrated cost savings, as the MCT + WF group had significantly more working days than patients who waited for treatment. Moreover, our findings for the entire sample suggest that MCT + WF led to substantial improvements in health-related quality of life that were maintained at 12 months after the end of treatment.

This RCT included patients with comorbid disorders (43%), was conducted at a typical outpatient clinic, and included relatively few treatment sessions, which supports the generalisability of our findings to similar public health settings. To the best of our knowledge, this is the first RCT to demonstrate large, combined reductions in anxiety and depression symptoms and sick leave among this population of patients.

The longitudinal sick leave data were obtained from a national registry, with no data attrition and supplemented by self-reported sick leave. Thus, this study directly complies with the OECD's strong recommendation for rigorous evaluations of new programs for individuals with mental health issues, such as including comparison groups and systematic data collection on long-term labour market outcomes.^5^ Norway's comprehensive welfare system provides full salary compensation to employees on sick leave, which may reduce motivation for patients and employers to expedite a RTW.^34^ Therefore, the national context of the highest sick leave rates and costs among OECD countries may have led to underestimation of the effects of MCT + WF compared to countries with other benefit systems.

Our results contrast with recent Scandinavian RCTs of work-focused cognitive behavioural therapy, which reported no impacts on sick leave rates for patients with CMD.^35^^,^^36^ Meta-analyses of RCTs of psychotherapy and work focus in CMD achieved similar non-significant findings or small effect sizes for symptoms and sick leave, with no significant long-term effects.^8^^,^^9^^,^^14^ This may indicate MCT is a more effective treatment for anxiety and depression than other psychotherapies. Evidence supports the superiority of MCT for treating anxiety and depression compared with CBT.^11^ The high effect sizes and recovery rates and sustained symptom reduction observed in our RCT are consistent with previous research on MCT in mental healthcare settings^11^^,^^12^^,^^37^ and extend the application of MCT to patients with anxiety and depression on sick leave. The high RTW rates observed in this study may also be attributed to the combination of the effectiveness of MCT and the flexibly tailored work focus, which included cooperation with the GPs who certified each patient's sick leave. However, the design of our RCT does not allow us to isolate the individual effects of the different components of the intervention.

Regardless of whether patients were randomised to immediate treatment or waiting, significant changes in the secondary outcomes, with large effect sizes, were observed between initial assessment and the end of treatment in both groups. Patients reported large reductions in dysfunctional metacognitions, which are considered important in the maintenance of anxiety and depression and are a central mechanistic target of MCT.^13^ From the perspective of RTW, positive effects were observed in RTW self-efficacy, which longitudinally predicts RTW.^22^ We also observed significant increases in resilience, suggesting patients gained greater access to personal and social resources essential for coping with adversity, which is associated with better mental health.^23^ Additionally, there was a significant improvement in self-rated health, a well-established predictor of general health status, including all-cause mortality.^24^ These findings suggest that the treatment positively affected a variety of secondary outcomes, and thus potentially reduces the risk of symptom relapse and subsequent sick leave. In addition, 19.1% of participants reported experiencing workplace bullying at assessment, which underscores the importance of thoroughly assessing the work environment during the work focus component of therapy.

Productivity cost analysis showed that the group receiving immediate MCT + WF experienced fewer sick leave days over the 12-month follow-up period compared to the patients who received delayed treatment. The calculated net reduction in productivity costs amounted to 6941 euros per patient for the 121 patients treated immediately, totalling a saving of 839,861 euros for this group after one-year. These findings highlight the economic drawbacks associated with waiting list practices in mental health services and demonstrate the delivery of prompt and efficacious treatment provides substantial economic and societal benefits compared to delayed treatment.^3^ The fact that our RCT achieved these encouraging findings within a typical healthcare setting indicates the broad applicability, scalability and potential of MCT + WF for real-world implementation.

The study has some limitations. First, the comprehensive Norwegian welfare system may influence the generalisability of our findings to other countries with different welfare systems. Nonetheless, the positive, long-lasting effects of MCT + WF in our Norwegian RCT is particularly interesting, as the OECD has identified Norway as a very challenging context for addressing sick leave.^5^ Second, we used a regular waiting list group as the comparator, and did not include an active control condition. However, this waitlist comparison represents the typical process of mental healthcare in developed countries, as patients usually wait several weeks for therapy after being referred. This increases feasibility in a real-world setting. Third, self-reported symptoms may be subject to response bias, particularly among individuals with depression and anxiety. Nevertheless, randomisation ensures that such biases are distributed non-systematically across groups. Fourth, a limitation of the study is that data were not available on whether participants in either group received additional medication or other mental health interventions during the 12 months follow up after MCT + WF. That said, given that participants in the treatment group were already engaged in an intensive intervention, it seems unlikely that they sought substantial additional care. If anything, participants in the waitlist control group may have accessed other forms of support during the waiting period, which would likely have reduced—rather than inflated—the observed treatment effect. Fifth, ethical approval precluded collection of data from non-participants, limiting assessment of potential selection bias. Furthermore, we did not analyse all costs related to sick leave; thus, the social savings resulting from MCT + WF are likely to be underestimated. Finally, there are some missing data in the exploratory secondary and longitudinal analyses over one year follow-up. Although these missing data occurred at random, this warrants careful interpretation of the results.

In conclusion, the substantial reduction in sick leave, large effect sizes, high symptom recovery rates, reduced productivity costs, low dropout rates, and sustained long-term symptom improvements observed in this naturalistic RCT suggest that MCT + WF may be a viable and effective approach for patients with CMD on sick leave. The results indicate that receiving MCT + WF early is beneficial for patients with anxiety and depression, as it may reduce the risk of prolonged sick leave and support faster symptom recovery. This study addresses both mental health and functional recovery, and thus responds to calls from the OECD^34^ and WHO.^6^ Replication studies are essential to validate the reliability and generalisability of this RCT, towards the goal of improving mental health, enhancing quality of life, and reducing the economic burden associated with anxiety and depression.

## Contributors

The study was conceptualised by RGHG, MH, KS, OH, KO, HMN, SER, SEL, RB, and AW, who also contributed to securing funding. OH, KS, RGHG, MH, HMN, SER, RB, SEL, and DW drafted the study protocol. The work-focused module was developed and adapted by MH, DW, RGHG, SEL, and RB, and supervision was provided to ensure fidelity. OH, served as chief investigator and supervised the study and the MCT fidelity. HDL and MGB coordinated and carried out data collection. KO, FA, RBTWG, SUJ, and OH contributed to writing the statistical analysis plan, while KO, FA, and OH performed the statistical analyses, accessed and verified the data. RGHG and OH drafted the manuscript, which was subsequently reviewed and critically revised by all authors. JS and KB were user representatives and contributed throughout all phases of the project, including manuscript preparation. All authors have read and approved the final version of the manuscript. The corresponding author confirms that all authors meet the criteria for authorship and that no eligible individuals have been excluded.

## Data sharing statement

Due to the sensitive nature of patient information and lack of approval for data sharing, the dataset from this study is not publicly available.

## Declaration of interests

All authors have completed the ICMJE uniform disclosure form at www.icmje.org/disclosure-of-interest/. AW is Director and shareholder of the Metacognitive Therapy Institute (MCTI) and has authored books and given workshops on metacognitive and cognitive-behaviour therapy. HMN is co-director of MCTI and has received royalties for book chapters, consulting and supervision fees, and honoraria for MCT presentations. RGHG and MH have received royalties for book chapters and standard honoraria for lectures on work-focused interventions. DW has received honoraria at standard institutional rates for lectures on common mental health problems, stress, sick leave, and work-focused therapy. SER and SUJ have received honoraria at standard institutional rates for lectures and presentations, unrelated to this manuscript. SER has also received travel reimbursement from the Norwegian Cancer Association and holds leadership positions in scientific societies. OH has received payment for presentations of MCT. All remaining authors declare no competing interests.
