# Supplementary material for: Metacognitive therapy and work-focus for patients with depression, anxiety or comorbid depression and anxiety on sick leave: a single-centre, open-label randomised controlled trial
Source: eClinicalMedicine. 2025 Nov 6;89:103613. doi: 10.1016/j.eclinm.2025.103613 (PMC12675033; doi:10.1016/j.eclinm.2025.103613)
Supplement: Appendix [file mmc1.pdf]

Supplementary appendices

This appendix formed part of the original submission and has been peer reviewed. We post it as supplied by the authors.

Supplement to: **Metacognitive Therapy and Work-Focus for Patients with Depression and Anxiety on Sick Leave: A Randomised Controlled Trial with A One-year Follow-Up**

Table of Contents

**Supplement**	Page
1: Outline of the protocol for Metacognitive Therapy	2
2: Description of the therapists' self-reported adherence checklists for MCT	3
3: Outline of the protocol for work focus	3
4: Description of the therapists' self-reported adherence checklists for work focus	8
5: Costs for economic data	9
6: Calculation of quality-adjusted life years (QALYs)	10
7: Search strategies	11
8: Table S 1 and Table S 2 Secondary outcome	17

Supplement 1. Outline of the protocol for Metacognitive Therapy

This study followed the protocol of metacognitive therapy (MCT) for treating anxiety and depression published by Prof. Adrian Wells in 2009. All therapists received advanced training in MCT® and weekly supervision by a specialist in MCT. In MCT, the treatment is based on a psychological model of information processing and self-regulation, namely the S-REF model [1-3]. The model proposes that the regulation of information processing in mental disorders is dominated by biased metacognitions that give rise to cognitive attentional syndrome (CAS) [4]. CAS consists of extended and repetitive thinking processes, typically in the form of worry and rumination, attentional focus involving threat monitoring, and dysfunctional coping strategies such as avoidance. MCT is based on a theory that anxiety and depression symptoms are maintained by perseverative CAS activities that interfere with adaptive self-regulation. The CAS is regulated by the metacognitive system that includes negative and positive beliefs about thinking. One key negative metacognition concerns the belief that thinking is uncontrollable and can also include beliefs that thoughts are dangerous. Positive metacognitive beliefs are related to the potential benefits of extended negative thinking (e.g. “I need to worry in order to be prepared”).

MCT emphasizes transdiagnostic mechanisms (metacognitions and the CAS) common across mental disorders and can be based on disorder-specific formulations representing such processes (most accurate) or a generic formulation.
The theory of MCT specifies that the maintaining factors are similar across anxiety and depression. As such, MCT is relevant both for anxiety and depression, in addition to comorbid disorders. The selection of the MCT case-formulation was based on the patient’s primary disorder. For example, patients with generalised anxiety (GAD) mainly worry, while patients with depression mainly ruminate. The content of these thought processes is different, but they are both perseverative thinking processes and can be addressed and formulated, e.g., the case-formulation can address both GAD and depression and is thereby suitable for comorbidity. Prior to a course of MCT, a general clinical assessment of the patient’s disorder is performed and an idiosyncratic case formulation is generated in a collaborative process between the patient and therapist. This is followed by socialization (familiarisation) to the model based on the case formulation. Treatment then focuses on challenging negative metacognitions related to the uncontrollability of cognition and subsequently, danger metacognitions. As part of this process patients are instructed through specific experiences designed to increase their awareness of their control of—and initiate new relationships with—negative mental thoughts. When negative metacognitions are modified, positive metacognitions are challenged. For example, the treatment encourages patients to challenge the potential benefits of worrying or rumination. Thereafter, CAS strategies related to maladaptive coping are challenged and banned. The final stages of MCT focuses on relapse prevention, by clarifying the differences between what maintained the disorder (old plan) and alternative self-regulatory strategies (new plan) that have been developed during treatment.

In the current study, the therapists included themes from work and work situations in the case formulation, during socialisation, when challenging negative and positive metacognitions, when modifying CAS activity (e.g., worry, avoidance), and when introducing return-to-work exercises. For example, many patients avoid thinking about return to work or worry about being able to manage tasks when they return to work. MCT is designed to help patients through exploration and experiential experiments to identify work-related thoughts as something that can be experienced without the need to anticipate danger or worry, and thus modify their beliefs about control.

Supplement 2: Description of the therapists' self-reported adherence checklists for MCT

Adherence to therapy protocols is important to ensure the correct methods are delivered in the correct sequence. The treatment manual for MCT [4] provides session-by-session content checklists for MCT. In the present study, the therapists self-rated their adherence to the appropriate checklists for each session.

At total of 117 MCT treatment plan checklists for depression, 89 for generalized anxiety, and two for post-traumatic stress disorder treatment were completed, representing a total 208 of 236 (88%) possible checklists to be assessed for adherence. Missing was attributed due to data collection being partly completed during the COVID-19 affecting the routines, and administrative errors at the clinic, or patient dropout. The therapists' mean self-rated adherence to the metacognitive components in the checklists was 89%

Table 1. Therapists' self-reported adherence checklists for MCT components completed during therapy. Values are mean percentage for all treatments for the ITT sample (*N* = 236)

Treatment components in MCT	Number completed/total number of adherence reports
Case conceptualisation	98.1%
Socialisation	99.5%
Therapist reported assigning and assessing relevant homework throughout the therapy	98.6%
Challenge negative metacognitions	99.5%
Challenge positive metacognitions	73.6%
Ban maladaptive coping strategies	86.5%
Relapse prevention with blueprint and old and new plan	66.3%
**Mean adherence for entire therapy**	**88.9%**

Supplement 3: Outline of the protocol for work focus

The protocol for work focus (WF) was designed as a set of themes that therapists should assess and address during therapy to support patients' return to work. The protocol was inspired by the Dutch study on W-CBT by Lagerveld and colleagues from 2012 and adapted to the Norwegian setting [5,6]. All therapists received advanced training and supervision from a psychologist licensed as specialized in clinical work psychology.

To maximize the relevance of this component of treatment, the work focus was personalized to each patient's unique circumstances, including their individual expectations, health conditions, work environment, and progress during therapy. For some patients, the primary challenge may lie in their mental health disorder(s). For other patients, their difficulties may stem from negative factors in the workplace that lead to risk factors for common mental disorders (CMD), such as low support, high workload, role conflicts, social unfairness, or bullying; in such cases, greater workplace adaptations or even a change in job may be necessary [7,8]. The central principle of work focus is to individualize the work themes in order to address each patient's unique mental health struggles and work-related challenges.

Assessing the work situation

Initial mapping

During the initial assessment sessions, each patient's work situation is mapped to provide the foundation for designing their specific work interventions. This involves understanding the patient's job type, workload, tasks, work-family conflict, autonomy, social environment, and relationships with colleagues and superiors. Identification of specific challenges, such as conflicts or bullying, is critical, as these factors may indicate the need for workplace adaptions, or even a change in job. If health issues affect functioning, the therapist explores which tasks are manageable and which task present difficulties due to mental health. This comprehensive mapping ensures that the interventions are specifically tailored to the patient's unique circumstances and goals.

Questions used to explore the work situation include:

- What are your specific work tasks, activities, and responsibilities?
- To what extent can you control the pace and organization of your work?
- Are there any current workplace problems (e.g., conflicts, harassment)?
- How is your relationship with colleagues and superiors?
- Which tasks do you find difficult or manageable, and why?
- What adjustments could make your work situation easier (e.g., reduced tasks, flexible hours)?
- Do you worry about losing your job?

Planning a return to meaningful work and evaluating sick leave

The therapist assesses the current degree of sick leave and evaluates whether it is appropriate for the patient to consider working at a higher percentage. For patients on full sick leave, the therapist explores the patient's thoughts about starting in a graded position and identifies potential barriers. Discussions may include possibilities for workplace adjustments and a realistic time frame for returning to work. It is also beneficial to discuss the employer's need for predictability regarding the patient's return to work. Therapists help patients reflect on the potential challenges and perspectives of the employer. This dialogue can serve as a foundation for improved communication between the patient and the workplace (see section on *Dialogue with the Workplace*). If suitable, a progressive "Return to Work Plan" can be outlined, detailing gradual reintegration (e.g., 30%, 50%, 80%).

Collaboration with the General Practitioner (GP) responsible for certifying sick leave

In Norway, GPs are responsible for issuing sick leave certifications. Effective communication and collaboration between the GP and therapist are critical for establishing shared goals and a consistent approach. In our RCT, each patient's GP was informed about the study when the patient consented to participate. After the second therapy session, the therapist, in collaboration with the patient, contacted the GP via letter to initiate a dialogue about the sick leave status and the return-to-work plan. In subsequent communication, the patient, therapist and GP agreed on a framework for ongoing collaboration between them throughout the treatment process.

Discussing the patient's experience of sick leave

The therapist explores the patient's experience of being on sick leave, identifying both positive and negative consequences of being away from work. This discussion serves three main purposes:

1. Evaluating possibilities for workplace and task adaptations
2. Identifying doubts or worries related to returning to work
3. Understanding external influences, such as advice from others to rest or avoid work until fully recovered (see section on *Assessing potential barriers*).

Assessing potential barriers

The therapist investigates the obstacles the patient perceives related to returning to work, and addresses concerns about these potential challenges, including:

- Anxiety about expectations or job performance
- Workplace bullying or conflicts (e.g., with colleagues or management)
- Worrying and avoidance behaviour, and fears about worsening symptoms upon returning to work
- High work pressure with low control
- Lack of recognition or support.

Key questions used to guide this exploration include:

- What makes it difficult for you to return to work?
- Do you have anxiety about expectations or job performance?
- Do you experience worrying and avoidance behaviour, and have fears about symptoms worsening upon returning?
- What do you think will contribute to improvement in your mental health and self-efficacy?
- What are the biggest obstacles or barriers to returning to work?
- What is needed to facilitate your return to work?
- Do you feel rest is essential for your recovery?
- Do you believe you need to be fully recovered before attempting to work again?

The therapist also examines how mental health symptoms affect functional abilities and identifies potential adjustments that may help.

Exploring resources

The therapist encourages the patient to reflect on aspects of work they miss and tasks they still enjoy or feel competent performing. This process helps to reconnect the patient with their professional identity and motivations. Additionally, the therapist maps out potential support from colleagues or workplace resources that could facilitate a return to work.

Dialogue with the workplace

The therapist helps the patient navigate how to approach communication with their workplace. It is often beneficial to initiate contact with the employer early, as prolonged sick leave tends to reduce the likelihood of returning to work. Many individuals find it challenging to discuss mental health issues openly, feeling uncertain or worrying about what to say and worried about the potential reactions of others in their workplace. Mental health challenges can unfortunately lead to stigma and discrimination in the workplace, which creates a dilemma: a patient being open may result in bias, while remaining silent may prevent access to necessary accommodations and support. Therefore, crafting an individualized information strategy is considered highly beneficial and often involves addressing key questions related to disclosing information about their mental health condition, the reasons for their sick leave, the adjustments they need, and the work that they can do:

- How open should I be?
- What should I say, to whom, and when?
- How much am I comfortable sharing, and in what way?
- What is the purpose of the information I provide?

Patients are encouraged to take an active role in communicating with their managers and colleges, which can contribute to confidence and return to work self-efficacy. If necessary, support from the human resources department of their workplace, union representatives, or external resources (e.g., Norwegian Labour and Welfare Administration) can be discussed. A tailored communication plan is developed to address patient-specific concerns and goals. A general recommendation is to share only what is necessary to create a supportive work environment (see section on accommodation) without the patient feeling the need to over-explain, apologize, or justify. Being clear and specific about needs for help in the workplace can lead to appropriate adjustments. It is equally important for the patient to communicate their strengths, highlighting what works well despite their mental health challenges and specifying which tasks they can and want to continue handling.

Adjustments at work

Adjustment is not solely about "removing" or simplifying work tasks; it is equally about retaining core responsibilities that provide a sense of meaning, accomplishment, and positive experiences. These tasks allow the patient to continue seeing themselves as a valuable resource, despite mental health challenges. The goal is to align the workplace demands with the patient's current capacity, to enable them to perform and succeed in their work.

When adjustments are challenging, it is important to explore what is realistically achievable. Every workplace is unique, and optimal adjustments may not always be possible. Therefore, discussions should focus on identifying potential solutions that balance the patient's needs with the workplace's framework. Key points to consider include:

- Is it necessary to reduce demands temporarily?
- How can demands be reduced? For example:
  - Fewer work tasks or responsibilities
  - Dividing tasks or delegating specific duties
  - Collaborating with a colleague or having a “sparring” partner
- Is there a need for clearer expectations?
  - Are job descriptions or performance expectations unclear? If so, clarify or specify tasks where uncertainty exists.
- Is additional training required?
  - Would a mentor help provide professional development and a sense of security?
- Is greater flexibility or self-control needed temporarily?
  - Examples include adjusting the pace of work, task prioritization, organization of the workday, or flexible working hours.
- Is extra social support needed?
  - For instance, regular check-ins with a manager, mentor, or support figure to provide guidance and constructive feedback.
- Which tasks does the patient feel confident in and wish to continue?
- What impact will the adjustments have on the workplace?
  - How feasible are the proposed changes, and what challenges might they present?
- What solutions would work best for both the patient and the workplace?

By addressing these considerations, it becomes easier to develop a practical and supportive adjustment plan that benefits both the patient and the workplace.

Strive for integration between work focus and therapy

Therapy and work focus should progress in an integrated manner to allow the patient to apply therapeutic strategies to real-world challenges and refine them as necessary. For example, work experiences can be integrated into case formulations or experiments in MCT to address worry, rumination, avoidance, and maladaptive coping strategies.

Prevention of sick leave

Towards the end of treatment, it can be invaluable to dedicate time to strategies to prevent future periods of sick leave due to work-related challenges, depression, or anxiety. This includes encouraging the patient to reflect on the potential mental health benefits of work, such as providing structure, social and professional stimulation, financial security, identity, and self-respect. It is worth noting that many individuals with mental health challenges remain in the workforce and perform well.

Even if the patient is considering a job change, returning to their current workplace while seeking other opportunities can still be beneficial. This allows the individual to practice alternative coping skills and potentially leave the job with a sense of accomplishment and dignity. Patients should also be prepared to possibly experience a temporary increase in symptoms upon returning to work, as this is expected and typically not harmful. Highlighting the long-term health benefits of work engagement can help patients manage these concerns.

Key questions to explore regarding workplace and mental health include:

- Develop an overview over the treatment and new strategies that promote healthy adaptation at work (consistent with plan B in the MCT manual)
- How can you discuss potential risk factors for mental distress at work (e.g., high workload, work-home conflict) with a manager or colleagues?
- What workplace adjustments might be helpful?
- How can collaboration with your general practitioner (GP) help prevent future sick leave?

At the conclusion of therapy, a discharge summary is prepared for all patients. This document includes the initial diagnosis, details of the therapy provided (e.g., MCT+WF), symptom and work status after treatment, and a prognosis. This summary is sent to the patient's GP to support ongoing care and prevent relapse.

Supplement 4. Description of the therapist's self-reported adherence checklists for work focus

Adherence to the work focus was measured through therapist self-reports, using two formats: a scheduled session checklist completed by the therapist (Table 2) and the therapist recording if work focus was applied during therapy or not (Table 3). Therapists evaluated themselves on whether each theme of the work focus described above was implemented in that session.

Given that this study was conducted in a regular outpatient clinic, the therapists treated more patients who were not included in the study than patients that were included. As such, the therapists faced the typical demands of a real-world clinical setting, which necessitated the development of a feasible solution for rating adherence that took into consideration their high caseloads. To enhance the likelihood of successful implementation, the adherence checklist for work interventions was co-developed with the therapists prior to the study's initiation. While these checklists lack the precision of video recordings, checklists are practical for use in naturalistic outpatient settings where therapists routinely manage heavy workloads. This approach may enhance the feasibility of testing whether the intervention can be generalized to other settings and patient samples, and thereby help to bridge the gap between research and clinical practice. During the therapy, therapists collected 213 out of 236 (90.3%) work focus checklists. The missing checklists were related to COVID-19 affecting the routines, and administrative errors at the clinic, or patient dropout. During the study a total of 208 out of 236 (88%) checklists were obtained and assessed for adherence. The therapists' mean self-rated adherence to the work focused themes in the checklists was 86% and 85 %, respectively (Table 2 and 3).

Table 2. Therapists' self-reported use of work focus interventions for each stage of treatment. Values are mean percentage for all treatments for the ITT sample (*N* = 236)

Number completed/total number of adherence reports	Work treatment protocol
	**Session 1**
97%	Inform patient about research and collaboration with GP (Letter)
89%	Provide knowledge and discuss relationship between sick leave, work, and mental health
97%	Current sick leave level and timeframe for RTW plan and recovery
	**Sessions 2-3**
89%	Resources related to the job (e.g., colleagues, boss, tasks)
90%	Barriers to returning to work (need for adjustments, dialogue, what is needed?)
74%	Feedback to GP in collaboration with the patient
	**Session 4 to last treatment session**
87%	Status update: barriers and resources at work now
80%	Is there a need for adjustments, dialogue with the manager, and other measures?
75%	Feedback to GP in collaboration with the patient
82%	Current sick leave level and timeframe for RTW plan and recovery

Table 3. Therapists' self-reported use of work interventions for the entire therapy. Values are mean percentage for all treatments for the ITT sample (*N* = 236)

Number completed/total number of adherence reports	Components of the work interventions summed up over total therapy
95%	Address experience of sick leave and work
88%	Education about sick leave
87%	Provide knowledge about work and mental health
94%	Exploration of barriers
87%	Exploration of resources related to work
86%	Address dialogue with the workplace
81%	Explore needs and opportunities for adjustments
85%	Aim for parallelism between work and therapy
78%	Evaluation of sick leave level and timeframe
71%	Use of behavioural experiments/therapeutic interventions related to work/job search

Supplement 5: Costs for economic data

Intervention cost for MCT and WF

Unit cost (summarised in Table 4) was calculated by adding up the total costs of the treatment resources at the psychiatric outpatient clinic, which receives approximately 1,000 patients per year, at Diakonhjemmet hospital. All costs were estimated for the financial year 2021.

Table 4. Intervention cost schema for the metacognitive therapy and work-focused intervention

Cost and unit	Unit cost 2021 NOK	Unit cost EUR
Salaries: wage costs for health personnel and administrative staff	13 271 250, -	1 161 689, -
Other costs/overheads:<br>-Management, commodities, hospital administration and estates<br>- Education, supervision, employees' social welfare	4 237 650, -	370 940, -
Total cost	17 508 900, -	1 532 628, -
**Mean cost per patient treated at the clinic**	**27 191, -**	**2 380, -**

The running expenses include all relevant clinical costs, as well as the extra costs for the therapists' advanced training in both MCT and work focus interventions.

Productivity cost

The productivity costs were estimated by multiplying the days of sick leave by the average national wage for men and women in Norway according to the human capital approach [9].

Table 5. Productivity costs estimated by summing up the cost of the total days on sick leave.

Productivity costs per sick leave day	NOK	EUR
Mean wage per. month in Norway	5 0790	4446
Mean national wage, women	47190	4131
Mean national wage, men	53710	4701
Mean wage, accounting for the ratio of genders 0,75*47190+0,25*53710	48820	4273
Per day	2547	223
Non-wage labour cost 40%	1019	89
**Total cost sick leave per workday**	**3566 (per workday)** [g]	**312**

Mean salary in Norway in 2021 from statistics Norway. Non-wage labour costs include the Norwegian employers' social cost of wages such as payroll tax and social security contributions. g = Includes salary and social costs (factor 1.4) (Statistics Norway).

Supplement 6: Calculation of quality-adjusted life years (QALYs)

We estimated the quality-adjusted life years (QALYs) of the immediate MCT+WF group and wait list control group from assessment to follow-up at 12 months after post-treatment. QALYs are a two-dimensional concept that combine both the quality and quantity of life [10]. We calculated QALYs based on the EQ-5D-5L and used the area-under-the-curve (AUC) method, which is a useful way to summarize the information from a series of measurements of one individual over time[12]. To measure AUC, health states were derived from individuals' responses to the EQ-5D-5L and converted into utility values using UK-specific value sets, reflecting the general population's preferences [11]. These value sets, representing utility weights for all possible health states, were used in line with national health economic evaluation guidelines. QALYs were then calculated by multiplying these utility weights by the time spent in each health state and aggregating the results to produce a total score adjusted for baseline [12,13].

To address the disparity in the follow-up times between the immediate treatment and waiting list groups, we employed interpolation to ensure equal time horizons across both groups. Generally, interpolation is considered the most accurate method as it relies on data within the observed range. To improve robustness, a sensitivity analysis was conducted using extrapolation.

In analysis of the EQ values, the total mean duration from assessment to 12 months follow-up after treatment was 15 months for the immediate MCT+WF group (treatment lasted on average 3 months) and 18 months for the waiting list group (as they waited approximately for 2 months before receiving treatment). The interpolation estimated EQ values for both groups based on the shortest timeframe (15 months). The extrapolation estimated EQ values for both groups based on the longest mean duration (18 months).

This analysis showed that the quality-adjusted life years (QALYs) increased from pre-treatment to 12-months follow-up after treatment for both groups in the main analysis; interpolation for the waiting list group resulted in a mean score of 1.18 (SD 0.19) for the waiting list group and 1.06 (SD 0.14) for the immediate MCT+WF group. Extrapolation conducted for a sensitivity analysis resulted in waiting list group score of 1.19 (SD 0.18) and 1.19 (SD 0.15) for the MCT+WF group. Both groups benefitted equally after receiving treatment in terms of quality-adjusted life years between assessment and 12-months post-treatment.

References

1. Wells A, Matthews G. Attention and emotion: A clinical perspective. Hove (UK). Lawrence Erlbaum. *Clinical Psychology & Psychotherapy* 1994; **2**: 134.
2. Wells A, Matthews G. Modelling cognition in emotional disorder: The S-REF model. *Behaviour research and therapy* 1996; **34**(11-12): 881-8.
3. Wells A, Fisher P, Myers S, Wheatley J, Patel T, Brewin CR. Metacognitive therapy in recurrent and persistent depression: A multiple-baseline study of a new treatment. *Cognitive therapy and research* 2009; **33**(3): 291-300.
4. Wells A. Metacognitive therapy for anxiety and depression, 2009. New yourk: Guilford press; 2009.
5. Lagerveld SE, Blonk RW, Brenninkmeijer V, Wijngaards-de Meij L, Schaufeli WB. Work-focused treatment of common mental disorders and return to work: a comparative outcome study. *J Occup Health Psychol* 2012; **17**(2): 220-34.
6. Gjengedal RG, Reme SE, Osnes K, et al. Work-focused therapy for common mental disorders: A naturalistic study comparing an intervention group with a waitlist control group. *Work* 2020; (Preprint): 1-11.
7. Harvey SB, Modini M, Joyce S, et al. Can work make you mentally ill? A systematic meta-review of work-related risk factors for common mental health problems. *Occupational and environmental medicine* 2017; **74**(4): 301-10.
8. Rugulies R, Aust B, Greiner BA, Arensman E, Kawakami N, LaMontagne AD, Madsen IE. Work-related causes of mental health conditions and interventions for their improvement in workplaces. *The Lancet* 2023; **402**(10410): 1368-81.
9. Robinson R. Cost-benefit analysis. *British Medical Journal* 1993; **307**(6909): 924-6.
10. Drummond MF, Sculpher MJ, Claxton K, Stoddart GL, Torrance GW. Methods for the economic evaluation of health care programmes: Oxford university press; 2015.

11. Legemiddelverk S. Guidelines for the submission of documentation for single technology assessment (STA) of pharmaceuticals. 2020.
12. Manca A, Hawkins N, Sculpher MJ. Estimating mean QALYs in trial-based cost-effectiveness analysis: the importance of controlling for baseline utility. *Health economics* 2005; **14**(5): 487-96.
13. van Reenen M, Janssen B. EQ-5D-5L user guide: basic information on how to use the EQ-5D-5L instrument. *Rotterdam: EuroQol Research Foundation* 2015; **9**.

Supplement 7: Search Strategies

Ovid MEDLINE(R) ALL <1946 to September 15, 2025>

1 Depression/ 173496
2 (depression or depression, emotional or depressive symptom or depressive symptoms or emotional depression or symptom, depressive or symptoms, depressive).mp. 580051
3 Depression/th [Therapy] 19722
4 Depression/rh [Rehabilitation] 702
5 Depression/di [Diagnosis] 25648
6 common mental disorder*.mp. 4548
7 exp Depressive Disorder, Major/ 43402
8 (depression, involutional or depressive disorder, major or depressive disorders, major or involutional depression or involutional melancholia or involutional paraphrenia or involutional paraphrenias or involutional psychoses or involutional psychosis or major depressive disorder or major depressive disorders or melancholia, involutional or paraphrenia, involutional or paraphrenias, involutional or psychoses, involutional or psychosis, involutional).mp. 59605
9 Depressive Disorder, Major/th [Therapy] 7790
10 Depressive Disorder, Major/px [Psychology]12367
11 exp Anxiety/ 129724
12 (angst or anxieties, social or anxiety or anxiety, social or anxiousness or hypervigilance or nervousness or social anxieties or social anxiety).mp. 370275
13 Anxiety/th [Therapy] 10618
14 Anxiety/rh [Rehabilitation] 274
15 Anxiety/di [Diagnosis] 11042
16 Anxiety Disorders Depression/di [Diagnosis] 0
17 anxiety disorder/th [Therapy] 7853
18 generalized anxiety disorder/th [Therapy] 1
19 exp Sick Leave/ 7249
20 (disability leave or leave, disability or leave, sick or sick leave or sick-list* or sick list*).mp. 11365
21 exp absenteeism/ 10186
22 absenteeism*.mp.15138
23 medical leave.mp. 389
24 "Leave of absence".mp. 284
25 sickness absence.mp. 3401
26 "long-term sick leave".mp.524
27 "long term sick leave".mp. 524
28 Employee Absenteeism.mp. 102
29 worker absenteeism.mp. 66
30 ("work absence" or "job absence" or "sickness absence" or "medical absence" or "illness absence" or "sick absence" or "work absenteeism" or "job absenteeism" or "sickness absenteeism" or "medical absenteeism" or "illness absenteeism" or "sick absenteeism" or "long term sick" or "long term sick leave" or "long-term sick*" or "long-term sick leave" or "disability leave" or "disability absence" or "disability absentee*").mp. 5702
31 Sick Leave/sn [Statistics & Numerical Data] 3068

32 Employee Leave Benefit*.mp. 1
33 work inability.mp. 42
34 ("work ability" or "workability").mp. 4381
35 exp "Return to Work"/ 4258
36 ("back to work" or back-to-work or "return to work" or return-to-work or "work, back to" or "work, return to" or "reemployment" or "re-employment").mp. 15287
37 ("return to work intervention*" or "return-to-work intervention*" or "back to work intervention*" or "back-to-work intervention* work site* intervention*" or "worksite* intervention*" or "job site* intervention*" or "jobsite* intervention*").mp. 362
38 "work focus".mp. 142
39 "work intervention*".mp. 695
40 "occupational intervention*".mp. 98
41 exp Cognitive Behavioral Therapy/ 42024
42 (behavior therapies, cognitive or behavior therapy, cognitive or behavioral therapies, cognitive or behavioral therapy, cognitive or behaviour therapies, cognitive or behaviour therapy, cognitive or cognition therapies or cognition therapy or cognitive behavior therapies or cognitive behavior therapy or cognitive behavioral therapies or cognitive behavioral therapy or cognitive behaviour therapies or cognitive behaviour therapy or cognitive psychotherapies or cognitive psychotherapy or cognitive therapies or cognitive therapy or psychotherapies, cognitive or psychotherapy, cognitive or therapies, cognition or therapies, cognitive or therapies, cognitive behavior or therapies, cognitive behavioral or therapies, cognitive behaviour or therapy, cognition or therapy, cognitive or therapy, cognitive behavior or therapy, cognitive behavioral or therapy, cognitive behaviour).mp. 45418
43 ("cognitive-behavioural treatment*" or "cognitive-behavioural therap*").mp. 6906
44 ("work-related cognitive-behavioral treatment*" or "work-related cognitive-behavioral therap*" or "work related cognitive behavioral therap*" or "work related cognitive behavioral treatment*").mp. 5
45 ("cognitive-behavioral treatment* as usual" or "cognitive behavioral treatment* as usual").mp. 3
46 "convergence dialogue meeting*".mp. 5
47 "Dialogue-Based Workplace Intervention*".mp. 2
48 (acceptance and commitment therapy).mp. 2551
49 exp "acceptance and commitment therapy "/ 1270
50 ("acceptance and commitment therapy" or "acceptance and commitment therap*" or "acceptance therap*" or "commitment therap*").mp. 2578
51 "workplace dialogue intervention*".mp. 4
52 "treatment as usual".mp. 7191
53 ("work-focused cognitive-behavioral therap*" or "work focused cognitive-behavioral therap*" or "work-focused cognitive behavioral therap*" or "work-focused cognitive-behavioral treatment*" or "work focused cognitive-behavioral treatment*" or "work-focused cognitive behavioral treatment*").mp. 6
54 ("work-directed intervention*" or "work directed intervention*").mp. 29
55 ("work-directed care" or "work directed care").mp. 6
56 Workplace Intervention*.mp. 1316
57 exp Psychotherapy/ or exp Behavior Therapy/ 234180
58 ("Metacognitive therap*" or "Meta-cognitive therap*" or "Metacognitive treatmen*").mp.332
59 Behavior Therapy/mt [Methods] 12653
60 Cognitive Behavioral Therapy/mt [Methods] 17222
61 (Acceptance and Commitment Therapy).mp. [mp=title, book title, abstract, original title, name of substance word, subject heading word, floating sub-heading word, keyword heading word, organism supplementary concept word, protocol supplementary concept word, rare disease supplementary concept word, unique identifier, synonyms, population supplementary concept word, anatomy supplementary concept word] 2551
62 exp Psychoanalytic Therapy/ 16184
63 (psychoanalytic therapies or psychoanalytic therapy or psychoanalytical therapies or psychoanalytical therapy or therapies, psychoanalytic or therapies, psychoanalytical or therapy, balint psychoanalytic or therapy, psychoanalytic or therapy, psychoanalytical).mp. 15732
64 Behavior Therapy/mt [Methods] 12653
65 ("Work-focused intervention*" or "Work focused intervention*").mp. 25
66 ("vocational reintegration" or "work reintegration" or "return to employment").mp. 382
67 1 or 2 or 3 or 4 or 5 or 6 or 7 or 8 or 9 or 10 or 11 or 12 or 13 or 14 or 15 or 16 or 17 or 18 795369
68 19 or 20 or 21 or 22 or 23 or 24 or 25 or 26 or 27 or 28 or 29 or 30 or 31 or 32 or 33 or 3429814

69 35 or 36 or 37 or 38 or 39 or 40 or 41 or 42 or 43 or 44 or 45 or 46 or 47 or 48 or 49 or 50 or 51 or 52 or 53 or 54 or 55 or 56 or 57 or 58 or 59 or 60 or 61 or 62 or 63 or 64 or 65 or 66 267803
70 67 and 68 and 69 830
71 exp Randomized Controlled Trial/ 648169
72 randomi?ed controlled trial*.mp. 965957
73 limit 70 to randomized controlled trial 187
74 71 or 72 967504
75 70 and 73 187
76 73 or 75 187

Embase <1974 to 2025 September 12>

1 Depression/ 597225
2 (depression or depression, emotional or depressive symptom or depressive symptoms or emotional depression or symptom, depressive or symptoms, depressive).mp. 1013019
3 Depression/th [Therapy] 32686
4 Depression/rh [Rehabilitation] 953
5 Depression/di [Diagnosis] 38498
6 common mental disorder*.mp. 5550
7 exp Depressive Disorder, Major/ 100148
8 (depression, involutional or depressive disorder, major or depressive disorders, major or involutional depression or involutional melancholia or involutional paraphrenia or involutional paraphrenias or involutional psychoses or involutional psychosis or major depressive disorder or major depressive disorders or melancholia, involutional or paraphrenia, involutional or paraphrenias, involutional or psychoses, involutional or psychosis, involutional).mp. 57547
9 Depressive Disorder, Major/th [Therapy] 9228
10 exp Anxiety/ 375229
11 (angst or anxieties, social or anxiety or anxiety, social or anxiousness or hypervigilance or nervousness or social anxieties or social anxiety).mp. 622238
12 Anxiety/th [Therapy] 1043
13 Anxiety/rh [Rehabilitation] 28
14 Anxiety/di [Diagnosis] 1611
15 Anxiety Disorders Depression/di [Diagnosis] 0
16 anxiety disorder/th [Therapy] 10529
17 generalized anxiety disorder/th [Therapy] 1586
18 exp Sick Leave/ 10386
19 (disability leave or leave, disability or leave, sick or sick leave or sick-list* or sick list*).mp. 10237
20 exp absenteeism/ or medical leave/ 30694
21 absenteeism*.mp. 25768
22 medical leave.mp. 10618
23 "Leave of absence".mp. 395
24 sickness absence.mp. 4067
25 "long-term sick leave".mp. 664
26 "long term sick leave".mp. 664
27 Employee Absenteeism.mp. 128
28 worker absenteeism.mp. 84
29 ("work absence" or "job absence" or "sickness absence" or "medical absence" or "illness absence" or "sick absence" or "work absenteeism" or "job absenteeism" or "sickness absenteeism" or "medical absenteeism" or "illness absenteeism" or "sick absenteeism" or "long term sick" or "long term sick leave" or "long-term sick*" or "long-term sick leave" or "disability leave" or "disability absence" or "disability absentee*").mp. 7424
30 Employee Leave Benefit*.mp. 2
31 work inability.mp. 71
32 ("work ability" or "workability").mp. 5046
33 exp "Return to Work"/ 12530
34 ("back to work" or back-to-work or "return to work" or return-to-work or "work, back to" or "work, return to" or "reemployment" or "re-employment").mp. 22947

35 ("return to work intervention*" or "return-to-work intervention*" or "back to work intervention*" or "back-to-work intervention* work site* intervention*" or "worksite* intervention*" or "job site* intervention*" or "jobsite* intervention*").mp. 450
36 "work focus".mp. 204
37 "work intervention*".mp. 926
38 "occupational intervention*".mp. 133
39 exp Cognitive Behavioral Therapy/ 45164
40 (behavior therapies, cognitive or behavior therapy, cognitive or behavioral therapies, cognitive or behavioral therapy, cognitive or behaviour therapies, cognitive or behaviour therapy, cognitive or cognition therapies or cognition therapy or cognitive behavior therapies or cognitive behavior therapy or cognitive behavioral therapies or cognitive behavioral therapy or cognitive behaviour therapies or cognitive behaviour therapy or cognitive psychotherapies or cognitive psychotherapy or cognitive therapies or cognitive therapy or psychotherapies, cognitive or psychotherapy, cognitive or therapies, cognition or therapies, cognitive or therapies, cognitive behavior or therapies, cognitive behavioral or therapies, cognitive behaviour or therapy, cognition or therapy, cognitive or therapy, cognitive behavior or therapy, cognitive behavioral or therapy, cognitive behaviour).mp. 91317
41 ("cognitive-behavioural treatment*" or "cognitive-behavioural therap*").mp. 10667
42 ("work-related cognitive-behavioral treatment*" or "work-related cognitive-behavioral therap*" or "work related cognitive behavioral therap*" or "work related cognitive behavioral treatment*").mp. 7
43 ("cognitive-behavioral treatment* as usual" or "cognitive behavioral treatment* as usual").mp. 4
44 "convergence dialogue meeting*".mp. 11
45 "Dialogue-Based Workplace Intervention*".mp. 2
46 (acceptance and commitment therapy).mp. 4888
47 exp "acceptance and commitment therapy "/ 4192
48 ("acceptance and commitment therapy" or "acceptance and commitment therap*" or "acceptance therap*" or "commitment therap*").mp. 4932
49 "workplace dialogue intervention*".mp. 4
50 "treatment as usual".mp. 12009
51 ("work-focused cognitive-behavioral therap*" or "work focused cognitive-behavioral therap*" or "work-focused cognitive behavioral therap*" or "work-focused cognitive-behavioral treatment*" or "work focused cognitive-behavioral treatment*" or "work-focused cognitive behavioral treatment*").mp. 12
52 ("work-directed intervention*" or "work directed intervention*").mp. 35
53 ("work-directed care" or "work directed care").mp. 9
54 Workplace Intervention*.mp. 1614
55 exp Psychotherapy/ or exp Behavior Therapy/ 348746
56 ("Metacognitive therap*" or "Meta-cognitive therap*" or "Metacognitive treatmen*").mp. 461
57 Behavior Therapy/ 49984
58 Cognitive Behavioral Therapy/ 39398
59 (Acceptance and Commitment Therapy).mp. 4888
60 exp Psychoanalytic Therapy/ 35091
61 (psychoanalytic therapies or psychoanalytic therapy or psychoanalytical therapies or psychoanalytical therapy or therapies, psychoanalytic or therapies, psychoanalytical or therapy, balint psychoanalytic or therapy, psychoanalytic or therapy, psychoanalytical).mp. 555
62 ("Work-focused intervention*" or "Work focused intervention*").mp. 35
63 ("vocational reintegration" or "work reintegration" or "return to employment").mp. 564
64 1 or 2 or 3 or 4 or 5 or 6 or 7 or 8 or 9 or 10 or 11 or 12 or 13 or 14 or 15 or 16 or 17 1298562
65 18 or 19 or 20 or 21 or 22 or 23 or 24 or 25 or 26 or 27 or 28 or 29 or 30 or 31 or 32 42009
66 33 or 34 or 35 or 36 or 37 or 38 or 39 or 40 or 41 or 42 or 43 or 44 or 45 or 46 or 47 or 48 or 49 or 50 or 51 or 52 or 53 or 54 or 55 or 56 or 57 or 58 or 59 or 60 or 61 or 62 or 63 409296
67 64 and 65 and 66 1543
68 exp Randomized Controlled Trial/ 1102782
69 randomi?ed controlled trial*.mp. 1482694
70 limit 67 to randomized controlled trial 394
71 68 or 69 1485213
72 67 and 71 495
73 70 or 72 495
74 limit 73 to "remove medline records" 187

APA PsycInfo <2002 to September 2025 Week 2>

1 Depression/ 0
2 (depression or depression, emotional or depressive symptom or depressive symptoms or emotional depression or symptom, depressive or symptoms, depressive).mp. 346710
3 common mental disorder*.mp. 2978
4 exp Major Depression/ 148292
5 (depression, involutional or depressive disorder, major or depressive disorders, major or involutional depression or involutional melancholia or involutional paraphrenia or involutional paraphrenias or involutional psychoses or involutional psychosis or major depressive disorder or major depressive disorders or melancholia, involutional or paraphrenia, involutional or paraphrenias, involutional or psychoses, involutional or psychosis, involutional).mp. 37433
6 exp Anxiety/ or exp Anxiety Disorders/ 101747
7 (angst or anxieties, social or anxiety or anxiety, social or anxiousness or hypervigilance or nervousness or social anxieties or social anxiety or Anxiety Disorder* or anxiet*).mp. 250852
8 exp Sick Leave/ 1656
9 (disability leave or leave, disability or leave, sick or sick leave or sick-list* or sick list*).mp. 2617
10 exp absenteeism/ 1687
11 absenteeism*.mp. 4944
12 medical leave.mp. 143
13 "Leave of absence".mp. 93
14 sickness absence.mp. 1323
15 "long-term sick leave".mp. 198
16 "long term sick leave".mp. 198
17 exp Employee Absenteeism/ or exp "Absenteeism (Employee)"/ or Employee Absenteeism.mp. 1720
18 worker absenteeism.mp. 16
19 ("work absence" or "job absence" or "sickness absence" or "medical absence" or "illness absence" or "sick absence" or "work absenteeism" or "job absenteeism" or "sickness absenteeism" or "medical absenteeism" or "illness absenteeism" or "sick absenteeism" or "long term sick" or "long term sick leave" or "long-term sick*" or "long-term sick leave" or "disability leave" or "disability absence" or "disability absentee*").mp. 1915
20 work inability.mp. 12
21 ("work ability" or "workability").mp. 1179
22 exp "Return to Work"/ 115
23 ("back to work" or back-to-work or "return to work" or return-to-work or "work, back to" or "work, return to" or "reemployment" or "re-employment").mp. 4001
24 ("return to work intervention*" or "return-to-work intervention*" or "back to work intervention*" or "back-to-work intervention* work site* intervention*" or "worksite* intervention*" or "job site* intervention*" or "jobsite* intervention*").mp. 183
25 "work focus".mp. 103
26 ("work intervention*" or "occupational intervention*").mp. 1017
27 exp Cognitive Techniques/ or exp Cognitive Therapy/ or exp Behavior Therapy/ or exp Cognitive Behavior Therapy/ 101147
28 (behavior therapies, cognitive or behavior therapy, cognitive or behavioral therapies, cognitive or behavioral therapy, cognitive or behaviour therapies, cognitive or behaviour therapy, cognitive or cognition therapies or cognition therapy or cognitive behavior therapies or cognitive behavior therapy or cognitive behavioral therapies or cognitive behavioral therapy or cognitive behaviour therapies or cognitive behaviour therapy or cognitive psychotherapies or cognitive psychotherapy or cognitive therapies or cognitive therapy or psychotherapies, cognitive or psychotherapy, cognitive or therapies, cognition or therapies, cognitive or therapies, cognitive behavior or therapies, cognitive behavioral or therapies, cognitive behaviour or therapy, cognition or therapy, cognitive or therapy, cognitive behavior or therapy, cognitive behavioral or therapy, cognitive behaviour).mp. 47053
29 ("cognitive-behavioural treatment*" or "cognitive-behavioural therap*").mp. 5877
30 ("work-related cognitive-behavioral treatment*" or "work-related cognitive-behavioral therap*" or "work related cognitive behavioral therap*" or "work related cognitive behavioral treatment*").mp. 5
31 ("cognitive-behavioral treatment* as usual" or "cognitive behavioral treatment* as usual").mp. 2
32 "convergence dialogue meeting*".mp. 4
33 "Dialogue-Based Workplace Intervention*".mp. 2
34 (acceptance and commitment therapy).mp. 4327
35 exp "acceptance and commitment therapy "/ 3365

36 ("acceptance and commitment therapy" or "acceptance and commitment therap*" or "acceptance therap*" or "commitment therap*").mp. 4353
37 "workplace dialogue intervention*".mp. 3
38 "treatment as usual".mp. 5136
39 ("work-focused cognitive-behavioral therap*" or "work focused cognitive-behavioral therap*" or "work-focused cognitive behavioral therap*" or "work-focused cognitive-behavioral treatment*" or "work focused cognitive-behavioral treatment*" or "work-focused cognitive behavioral treatment*").mp. 3
40 ("work-directed intervention*" or "work directed intervention*").mp. 12
41 ("work-directed care" or "work directed care").mp. 0

Supplementary 8: Secondary Outcome

**Secondary outcome**		**n**	**Assessment (SE)**	**Post waiting/End of treatment (SE)**	**Time x group effect coefficient (*p*-value)**	**Time x group effect 95% CI**	***d***
EQ-5D-5L							
	Waiting list	115	0.59 (0.02)	0.65 (0.02)	0.18 (<0.0001)	[0.12, 0.23]	0.28
	MCT+WF	121	0.57 (0.02)	0.81 (0.02)			1.09
	Group difference	236	-0.02 (0.03)	0.16*(0.03)			
RTW-SE							
	Waiting list	115	2.69 (0.08)	2.89 (0.09)	1.47 (<0.0001)	[1.22, 1.73]	0.16
	MCT+WF	121	2.71 (0.08)	4.39 (0.09)			1.79
	Group difference	236	0.03 (0.11)	1.50*(0.13)			
RSA							
	Waiting list	115	4.31 (0.07)	4.50 (0.08)	0.63 (<0.0001)	[0.47, 0.80]	0.23
	MCT+WF	121	4.25 (0.07)	5.06 (0.08)			0.98
	Group difference	236	-0.06 (0.10)	0.57*(0.11)			
MCQ-30							
	Waiting list	115	62.42 (1.19)	61.23 (1.23)	-15.58 (<0.0001)	[-19.86, -13.15]	0.09
	MCT+WF	121	63.94 (1.16)	47.13 (1.26)			1.01
	Group difference	236	1.49 (1.66)	-14.10*(1.76)			
SHC							
	Waiting list	115	25.76 (0.99)	24.07 (1.03)	-10.20 (<0.0001)	[-12.62, -7.78]	0.14
	MCT+WF	121	26.57 (0.96)	14.69 (1.06)			1.07
	Group difference	236	0.82 (1.37)	-9.38*(1.48)			
SAPAS-SR	Waiting list	115	2.78 (0.13)	2.62 (0.15)	-0.04 (0.16)	[-0.35, 0.28]	0.11
	MCT+WF	121	2.78 (0.12)	2.59 (0.14)			0.13
	Group difference	236	0.00 (.018)	-0.04 (0.20)			
AUDIT	Waiting list	115	4.92 (0.35)	4.53 (0.36)	-0.33 (0.28)	[-0.87, 0.22]	0.12
	MCT+WF	121	5.32 (0.34)	4.60 (0.35)			0.19

	Group difference	236	0.39 (0.49)	0.07 (0.51)

Secondary outcomes were measured at the last treatment session in the immediate MCT+WF group (median number of sessions = 10, IQR 7–13) and after a mean waiting period of 9.14 weeks in the waiting list group.

Increases in the RTW-SE, RSA, and EQ-5D-5L scores indicate improvement and reductions in the MCQ-30 and SHC scores indicate improvement.

* $p < 0.05$. RTW-SE=Return-to-Work Self-Efficacy questionnaire. RSA=Resilience Scale for Adults. MCQ-30=Metacognitions Questionnaire 30. SHC=Subjective Health Complaints questionnaire. EQ-5D-5L=Health-Related Quality of Life. EuroQol five dimensions descriptive system. SAPAS-SR=The Standardised Assessment of Personality Abbreviated Scale – Self report. AUDIT= Alcohol Use Disorder Identification Test.

Table S1: **Multilevel modelling with planned contrast of secondary outcomes at the end of treatment/after treatment in the immediate MCT+WF group (n = 121) and post-waiting in the waiting list group (n = 115).**

Secondary outcomes		Model-predicted means					
		n	**End of - treatment (SE)**	**6-months follow-up (SE)**	**12-months follow-up (SE)**	**Difference in change over time, Time x group effect coefficient (p-value)**	**Time x group effect 95% CI**
**BDI-II**							
	Waiting list	115	11.49 (0.91)	11.24 (0.98)	10.03 (1.07)	-2.46 (0.050)	[-4.92, -0.02]
	MCT+WF	121	13.27 (0.88)	9.88 (0.99)	9.35 (1.04)		
	Group difference	236	1.78 (1.26)	-1.35 (1.39)	-.68 (1.49)		
**BAI**							
	Waiting list	115	6.99 (0.77)	7.28 (0.82)	7.20 (0.88)	-1.18 (0.21)	[-3.04, 0.67]
	MCT+WF	121	8.54 (0.74)	7.44 (0.82)	7.57 (0.85)		
	Group difference	236	1.55 (1.07)	.16 (1.16)	.37 (1.22)		
**EQ-5D-5L**							
	Waiting list	115	0.84 (0.01)	0.84 (0.01)	0.84 (0.02)	0.03 (0.25)	[-0.02, 0.07]
	MCT+WF	121	0.82 (0.01)	0.82 (0.01)	0.85 (0.02)		
	Group difference	236	-0.03 (0.02)	-0.02 (0.02)	0.00 (0.02)		
**RTW-SE**							
	Waiting list	115	4.36 (0.10)	4.51 (0.10)	4.51 (0.12)	0.24 (0.12)	[-0.06, 0.55]
	MCT+WF	121	4.44 (0.09)	4.75 (0.11)	4.84 (0.11)		
	Group difference	236	0.08 (0.14)	0.24 (0.15)	0.32 (0.16)		
**RSA**							
	Waiting list	115	5.21 (0.09)	5.06 (0.09)	5.20 (0.10)	0.12 (0.36)	[-0.14, 0.38]
	MCT+WF	121	5.11 (0.09)	5.16 (0.09)	5.23 (0.10)		
	Group difference	236	-0.10 (0.12)	0.10 (0.13)	0.02 (0.14)		
**MCQ-30**							
	Waiting list	115	44.11 (1.08)	43.01 (1.14)	44.38 (1.32)	-2.42 (0.108)	[-5.37, 0.53]
	MCT+WF	121	47.39 (1.07)	44.32 (1.17)	45.24 (1.31)		
	Group difference	236	3.28 (1.52)	1.32 (1.63)	.86 (1.85)		
**SHC**							
	Waiting list	115	12.98 (1.03)	13.11 (1.05)	13.74 (1.11)	-2.45 (0.030)	[-4.67, -0.24]

MCT+WF	121	14.35 (1.02)	13.98 (1.08)	12.67 (1.11)
Group difference	236	1.37 (1.45)	0.87 (1.51)	-1.07 (1.57)

Secondary outcomes were measured at the last treatment session in the immediate MCT+WF group and at last treatment session in the waiting list group with delayed MCT+WF.

Reductions in the BDI-II, BAI, MCQ-30, and SHC scores indicate improvement and increases the RTW-SE, RSA, and EQ-5D-5L scores indicate improvement. BDI-II=Beck Depression Inventory II. BAI=Beck Anxiety Inventory. RTW-SE=Return-to-Work Self-Efficacy questionnaire. RSA=Resilience Scale for Adults. MCQ-30=Metacognitions Questionnaire 30. SHC=Subjective Health Complaints. EQ-5D-5L= Health-Related Quality of Life. EuroQol five dimensions descriptive system.

Table S2: **Model predicted means showing the difference in change over time for the secondary outcomes from the end of treatment to 6- and 12-months follow-up after treatment for the immediate MCT+WF group (n = 121) and the waiting list group (n = 115).**
