# Supplementary material for: Metacognitive therapy and work-focus for patients with depression, anxiety or comorbid depression and anxiety on sick leave: a single-centre, open-label randomised controlled trial
Source: eClinicalMedicine. 2025 Nov 6;89:103613. doi: 10.1016/j.eclinm.2025.103613 (PMC12675033; doi:10.1016/j.eclinm.2025.103613)
Supplement: Supplementary Material [file mmc2.pdf]

The following translations in Norwegian were submitted by the authors and we reproduce them as supplied. They have not been peer reviewed. Our editorial processes have only been applied to the original abstract in English, which should serve as reference for this manuscript.

Sammendrag

Bakgrunn:

Arbeid er en avgjørende faktor for helse, men behandling av psykiske lidelser har vist begrenset effekt på retur til arbeid ved depresjon og angst. Vi undersøkte effekten av metakognitiv terapi kombinert med arbeidsfokus (metacognitive therapy + work focus; MCT+WF) på retur til arbeid og symptomer på angst og depresjon hos pasienter som var sykmeldte.

Metode

Denne enkelt-senter, åpne, randomiserte kontrollerte studien ble gjennomført ved en poliklinikk på Diakonhjemmet sykehus i Norge. Deltakere var voksne pasienter som var sykmeldte med depresjon og/eller angst. Pasienter med alvorlige psykiske lidelser eller rusmisbruk ble ekskludert. Deltakere ble tilfeldig fordelt ved hjelp av en blokk-randomisering generert av ett dataprogram, stratifisert etter kjønn og grad av sykmelding, til enten umiddelbar MCT+WF eller utsatt MCT+WF etter 8-12 uker på venteliste. De som vurderte utfallet var blindet. Primære utfallsmål var depresjon (BDI-II) og angst (BAI) og retur til arbeid på 12 uker. Sykmeldingsdata ble hentet fra nasjonale register og selvrapport; symptomer ble målt var selvrapport. Analysene fulgte prinsippet om intention-to- treat analyse og inkluderte alle randomiserte pasienter. Studien var registrert hos ClinicalTrials.gov (NCT03301922; https://clinicaltrials.gov/study/NCT03301922).

Resultater

I perioden 11. september 2017 til 17. november 2020 ble 236 pasienter inkludert og randomisert til umiddelbar MCT+WF (n=121) eller venteliste (n=115). Etter 12 uker viste en logistisk regresjonsanalyse av register data signifikant høyere retur til arbeid i den umiddelbare MCT+WF gruppen (39%; 47/121) sammenliknet med venteliste gruppen (20%; 23/115; OR=2·39, 95% CI 1·32–4·32; p=0·0040), i tråd med selvrapportert retur til arbeid 42% (51/121) versus 18% (20/114); OR=3.44, 95% CI: [1.87, 6.35], p<0.0001). Multinivåmodeller viser større reduksjon i angst (interaksjonskoeffisient tid x gruppe = –8·35, 95% CI –10·61 to –6·09; p<0·0001) og depresjon (–10·84, 95% CI –13·25 to –8·44; p<0·0001) i umiddelbar MCT+WF versus venting.

Implikasjoner

Umiddelbar MCT+WF resulterte i signifikant høyere retur til arbeid og en stor reduksjon i symptomer for depresjon og angst sammenliknet med venting. Generaliserbarheten kan være begrenset av det norske velferdssystem, men styrker inkluderer bruk av registerdata og en

naturalistisk poliklinisk setting. Funnene antyder at MCT+WF kan integreres i psykisk helsevern.
